# Integrated Dialysis Nursing Intervention for Ameliorating Fatigue in Hemodialysis Patients

**DOI:** 10.4314/ejhs.v34i5.7

**Published:** 2024-09

**Authors:** S Yogalakshmi, D Sasikala, Santosh Varughese, Vasanthakumari Sundararajan

**Affiliations:** 1 Tamil Nadu Dr MGR Medical University, Guindy, Chennai; 2 Department of Fundamental Nursing, Tamil Nadu Dr MGR Medical University, Guindy, Chennai; 3 Department of Nephrology, Christian Medical College, Tamil Nadu Dr MGR Medical University, Guindy, Chennai; 4 School of Nursing and Midwifery, Institute of Health Sciences, Wallaga University, Ethiopia

**Keywords:** Integrated nursing intervention, Dialysis, Fatigue, Hemodialysis, Aerobic exercise, Chronic kidney disease

## Abstract

**Background:**

Fatigue is a pervasive and debilitating symptom among hemodialysis patients, severely impacting their quality of life and ability to participate in social activities. Dialysis nurses are pivotal in alleviating these effects through physical exercise. This study aims to evaluate the effectiveness of an integrated dialysis nursing intervention in reducing fatigue among hemodialysis patients.

**Methods:**

A quasi-experimental time series design was employed, involving 295 hemodialysis patients (148 in the experimental group and 147 in the control group) selected through consecutive sampling from two dialysis units in Chennai. Baseline fatigue was assessed in both groups. The experimental group received the integrated dialysis nursing intervention, including 15-minute sessions of aerobic exercises three times a week for eight weeks. The control group continued with routine care. Fatigue levels were reassessed at the end of the fourth and eighth weeks. Data were analyzed using SPSS version 20.

**Results:**

The study revealed a significant reduction in fatigue scores in the experimental group compared to the control group, with p < 0.001 in post-test I and II. The experimental group showed greater improvement than the control group, with p < 0.05.

**Conclusions:**

The integrated dialysis nursing intervention significantly reduced fatigue in hemodialysis patients. Incorporating this approach into routine intradialytic care can enhance fatigue management and improve patients' quality of life.

## Introduction

Chronic kidney disease (CKD) is a major global health issue, progressing to end-stage renal disease (ESRD) and requiring dialysis. In India, with an increased life expectancy reported at 66 years as of 2013, the prevalence of lifestyle diseases such as diabetes and hypertension has also risen (1). Approximately 40–60% of CKD cases are attributable to these conditions. Hemodialysis is a crucial treatment, with an estimated 175,000 chronic dialysis patients in India as of 2018, and a 10–20% annual increase in new patients (2, 3). Common symptoms among CKD patients include fatigue, depression, anxiety, muscle cramps, and restless leg syndrome (RLS) (1, 4–6). Fatigue affects 60–97% of hemodialysis patients, impairing their quality of life and making them feel exhausted. Contributing factors include dietary restrictions, stress, and dialysis effects on the body (7–8). While exercise has been shown to improve oxidative metabolism and muscle mass, it remains underutilized in dialysis units in India (12). Exercise can enhance physical and psychological well-being, improve kidney function, and preserve muscle integrity (13). Despite its benefits, exercise is rarely practiced due to lack of awareness among nurses and patients. This study aims to address this gap by evaluating the impact of integrated dialysis nursing interventions on fatigue in hemodialysis patients.

## Materials and Methods

**Study design and setting**: A quasi-experimental time series design was used to assess the effectiveness of integrated dialysis nursing interventions on fatigue in hemodialysis patients at a selected hospital in Chennai.

**Sample**: The sample size, estimated based on prior research (17), was 168 per group with a 20% attrition adjustment, resulting in 300 patients (150 per group). Inclusion criteria included patients aged 20–59 years, fluent in English or Tamil, and on hemodialysis for at least three months. Exclusion criteria included inability to follow exercise regimens, unstable hemodynamic parameters, recent angina, contraindications to exercise, and certain blood and health conditions. A total of 295 participants (148 experimental, 147 control) completed the study.

**Measurements**: The 10-item Fatigue Assessment Scale (18) was used to measure fatigue, with scores ranging from 10 to 50 and categorized as nonfatigue (≤50%), fatigue (51–75%), or extreme fatigue (>76%). The scale was validated and pretested for reliability (Cronbach alpha 0.83).

**Ethical considerations**: The study adhered to the Helsinki Declaration and received institutional ethics committee approval. Informed consent was obtained from all participants.

**Data collection procedure**: Data were collected from February to May 2021. Baseline fatigue levels were assessed, and the experimental group underwent aerobic exercises during the first two hours of each dialysis session for eight weeks. Post-test assessments were conducted at weeks 4 and 8.

**Data analysis**: Data were analyzed using SPSS version 20, with descriptive and inferential statistics including t-tests, RM ANOVA, post hoc analyses, and ANCOVA.

## Results

The results revealed that there were no appreciable differences in clinical and demographic factors between the experimental and control groups except for creatinine and haemoglobin levels (Tables 1 and 2). Approximately 50% of the haemodialysis patients in both groups were married and aged between 51 and 60 years. Within both categories, the distributions of both genders were almost equal. Almost two-thirds of the patients were married, nonvegetarians, graduates earning more than Rs. 25,000 a month and did not regularly follow any exercise regimen. Nearly two-thirds of the patients had a family history of ESRD, over half of the patients had comorbidities such as diabetes mellitus or hypertension, and approximately one-third of the patients had an unknown cause for their chronic kidney disease. Additionally, more than one-third of the plants in the experimental and control groups had been on HD for more than three years and weighed more than 60 kg of dry weight.

**Table 1:** Frequency and percentage distribution of demographic variables of hemodialysis patients

Variables and Categoty		Control (n=147)	Experimental (n=148)	p
Age in years	21-30 years	21	24	0.90
	31-40 years	23	26	
	41-50 years	41	37	
	51-60 years	62	61	
Gender	Male	77	82	0.60
	Female	70	66	
Residence	Rural	30	32	0.80
	Semi urban	30	34	
	Urban	87	82	
Dietary habit	Vegetarian	62	57	0.51
	Non Vegetarian	85	91	
Education	Illiterate	10	10	0.98
	Primary/middle	23	21	
	High school/HSC	19	18	
	Graduate and above	95	99	
Occupation	Home maker	26	21	0.92
	Unskilled	9	10	
	Semi-professional	78	79	
	Professional	12	15	
	Retired	22	23	
Income in rupees	<Rs. 10000	19	18	0.79
	Rs. 10,001-15,000	32	26	
	>Rs. 15,000	96	104	
Marital status	Unmarried	36	28	0.52
	Married	87	100	
	Widowed	16	13	
	Divorced	8	7	
regular exercise	Yes	51	58	0.42
	No	96	90	
Primary caregiver	Self	44	34	0.40
	Spouse	65	64	
	Son	17	22	
	Daughter in law	21	28	

**Table 2:** Frequency and percentage distribution of clinical variables of hemodialysis patients

Variables and Category		Control (n=147)	Experimental (n=148)	p
Comorbidities	Diabetes mellitus	24	22	0.94
	Hypertension	15	14	
	Both	80	86	
	Other renal	28	26	
Family history of	Yes	96	92	0.57
ESRD	No	51	56	
Cause for kidney	Diabetes mellitus	24	22	0.93
disease	Hypertension	39	35	
	polycystic kidney disease	15	19	
	Unknown etiology	37	38	
	Any other cause	32	34	
Duration of ESRD	< 1 year	34	34	0.65
	1-3 years	36	32	
	3-5 years	20	28	
	.> 5 years	57	54	
Duration of HD	.<6 months	14	11	0.93
	6 months-1 year	21	23	
	1-2 years	26	26	
	2-3 years	30	35	
	.>3 years	56	53	
Dry weight	31-40kg	14	13	0.96
	41-50kg	34	33	
	51-60kg	49	47	
	>60kg	50	55	
HD treatment per	Thrice in a week	121	119	0.67
week	Twice in a week	26	29	
Sr.urea		92.42±38.30	84.75±31.67	0.062
Sr.Creatinine		6.85±1.12	6.38±1.44	0.002
Sr.Potassium		4.90±0.71	4.77±0.78	0.154
Sr. Hemoglobin		8.39±0.62	8.64±0.73	0.002

More than 85% of the CKD patients reported fatigue, and one-third reported severe fatigue, with variations in the 4^th^ and 8^th^ weeks. The degree of variation was greater in the experimental group (Table 3).

**Table 3:** Comparison of level of fatigue during pretest, posttest-I and posttest-II among experimental and control group

Group	Level of fatigue	Assessments						P

		Pretest		Posttest-I		Posttest-II		

		n	%	n	%	n	%	
**Experimental** **(n =148)**	Non fatigue	12	8.11	38	25.68	69	46.62	0.001
	Fatigue	52	35.14	71	47.97	58	39.19	
	Extreme fatigue	84	56.76	39	26.35	21	14.19	
**Control (147)**	Non fatigue	19	12.93	22	14.97	25	17.01	0.16
	Fatigue	58	39.46%	59	40.14	62	42.18	
	Extreme fatigue	70	47.62	66	44.90	60	40.82	

**Figure 1:**
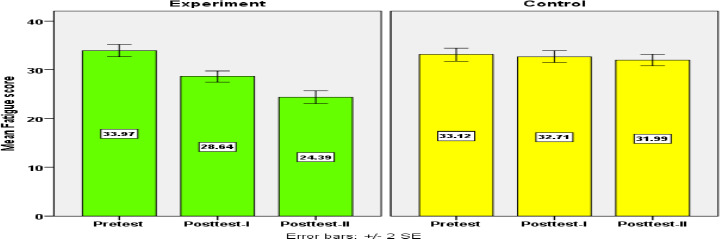
Comparision of fatigue score between experimental and control group during pretest, posttest-I and posttest-II

Repeated-measures ANOVA and post hoc analysis revealed that there was no discernible decrease in fatigue scores in the control group between the pretest and posttest. However, in the experimental group, there was a significant difference in the mean fatigue levels between pretest and posttest I and II (p < 0.001). The post hoc analysis demonstrated a decrease in posttest II scores, thus illustrating the long-term beneficial effects of the intervention (Table 4 and Figure 1).

**Table 4:** Comparison of pretest, posttest I and II fatigue scores in control and experimental group of haemodialysis patients

Assessment	Control (147)	Experimental (148)	P
Pre-test	33.12 ± 8.36	33.12 ± 7.47	0.231
Post-test-I	32.70 ± 7.58	32.71 ± 7.03	0.731
Post-test-II	31.98 ± 6.91	31.99 ± 7.80	0.020

**Post Hoc Analysis**	**Mean Difference**		

Pre-test vs post-test-I	0.415	5.32	
	p=1.000	p=0.001	
Pre-test vs Post-test-II	1.136	9.57	
	p=0.337	p<0.001	
Post-test-I Vs post-test-II	0.721	4.25	
	p=0.770	p<0.001	

Independent samples t tests revealed that there were no significant differences in pretest fatigue scores between the groups. The reduction in posttest fatigue scores in the experimental group was significantly greater than that observed in the control group (p<0.05). With respect to the itemwise scores, the reduction percentages ranged from 15.2% to 25.6% in the experimental group, with a mean reduction of 19.16%, which is higher than the 2.26% reported in the control group (Table 5). The multivariate analysis revealed that after controlling for the effects of pretest haemoglobin and urea levels, the intervention had a significant effect on reducing fatigue (21%; P<0.001).

**Table 5:** Item wise Comparison of Mean Percentage of Pre and Post-Intervention II Fatigue Score among Experimental Group

S. no	Items	Control group (n=147)			Experimental group (n=148)		

		Pretest	Post-test-II	Diff	Pretest	Post-test-II	Diff
1	I am bothered by fatigue.	61.8	60.4	1.4	63.4	48.2	15.2
2	I get tired very quickly	72.4	70.8	1.6	73.8	55.2	18.6
3	I don't do much during the day	73.0	70.6	2.4	72.8	53.4	19.4
4	I have enough energy for every day	67.8	64.8	3.0	70.0	45.4	24.6
5	I generally don't find life enjoyable	58.2	56.2	2.0	60.6	44.8	15.8
6	I have problems starting things	73.8	69.6	4.2	76.0	50.4	25.6
7	I have problems thinking clearly	70.4	66.8	3.6	71.8	50.0	21.8
8	I feel no desire to do anything	70.8	67.6	3.2	73.2	52.4	20.8
9	Mentally I feel exhausted	51.8	52.8	-1.0	52.6	38.2	14.4
10	When I am doing something, I can concentrate quite well	62.4	60.2	2.2	65.2	49.8	15.4
	**Total**	66.24	63.98	2.26	67.94	48.78	19.16

## Discussion

The current study aimed to determine the effects of integrated dialysis nursing interventions, which include tailored range-of-motion exercises for the upper extremity, on fatigue. Fatigue is most commonly reported and acknowledged worldwide as a crippling symptom [19] by patients with CKD. It is considered one of the most prevalent and incapacitating symptoms of CKD [20]. In this study, approximately 85% of CKD patients reported feeling fatigued, and one-third reported having severe fatigue, with variations in the 4^th^ and 8^th^ weeks after the intervention. Fatigue in CKD is related to complex factors such as reduced oxygen delivery, increased dependence on anaerobic metabolism, and lactic acidosis, with consequential depressive symptoms, sarcopenia, chronic fatigue, and atrophy of skeletal muscles [21]. Regular exercise improves the physiological and psychological symptoms of ESRD; according to recent studies, exercise and physical activity have been demonstrated to reduce fatigue. The exercises are advised as successful interventions to enhance physical function and fatigue because of their positive aspects of improved cardiovascular and mental health status [22]. Our study results revealed an greater decrease in fatigue at the posttest I assessment in the experimental group than in the control group. The post hoc analysis revealed a further decrease in fatigue at the posttest II assessment, thus indicating the long-term beneficial effects of the intervention. Motivation and counselling are vital in actively participating in exercise programs for individuals with ESRD. Social relationships, including family relationships, are reported to be inversely related to depression due to illness (23). This explains the crucial role of family members in motivating haemodialysis patients for strong endurance in adhering to the exercise regimen. These results were corroborative with similar study results indicating a noticeable reduction in fatigue after the intradialytic aerobic exercise programme. The flow of urea from the tissue into the vascular compartment appears to be enhanced by dialysis exercise, which also seems to increase blood flow to the muscles while increasing the capillary surface area (24) by increasing muscle artery width. Therefore, we can anticipate an improvement in the quality of life of patients receiving haemodialysis because of increased blood circulation, improved perfusion, improved toxin elimination, increased muscular strength, and ultimately decreased levels of fatigue and impotence. (25). Another study conducted by Samuel reported a statistically significant difference in fatigue scores between the two groups (F=10.513, P < 0.001). Post hoc comparisons between groups revealed no significant difference at baseline or at 12 weeks, but a significant difference (P = 0.001) at 24 and 36 weeks was noted between the experimental and control groups (26). The study results were also in accordance with those of an evaluative study by Tamilmozhi et al., who reported a significant reduction in the level of fatigue (p<0.05) (27).

The findings were in line with those of Malini et al., who reported that the Kt/V (dialysis adequacy) and urea reduction ratio increased and that the fatigue level decreased in the treatment group, whereas these variables remained unchanged in the control group. Substantial mean differences in Kt/V, the urea reduction ratio, and fatigue were reported between the groups at the end of Week 8 (28), and exercise training is likely to improve functional capacity (29). Studies have reported that intradialytic aerobic resistance combined with exercise is the most effective method compared with routine care (30). In this study, the control and experimental groups presented significant differences in haemoglobin levels. Although a notable improvement in fatigue was reported in the experimental group, the change in the fatigue score of both groups was considered marginal with respect to haemoglobin. Additionally, these findings suggest that to further reduce fatigue, therapies aimed at increasing haemoglobin levels may be needed. Erythropoiesis-stimulating medicines that target increased haemoglobin levels may improve fatigue; however, their use as a stand-alone treatment for fatigue has potential cardiovascular side effects. The current guidelines advise careful customization of haemoglobin targets for individuals at low cardiovascular risk who nevertheless feel fatigued or have functional limitations even at a haemoglobin level of 10 g/dl (31). Studies have reported a positive significant relationship between emotional intelligence and self-efficacy, and the reinforcement of emotional intelligence facilitates learning. (32, 33). This implies the necessity of nurturing continual psychological support and conducting awareness programs on the benefits and positive effects of an integrated customized nursing approach for better therapeutic compliance to alleviate fatigue and improve physical activity. This reduces mortality due to cardiac complications among CKD patients. Aerobic exercise lasting longer than 20 minutes and performed within the first two hours of haemodialysis at a frequency of less than 12 sessions is considered beneficial for reducing fatigue. Hence, the implementation of a educational nursing program as a low-cost, effective, and nonaggressive intervention can reduce fatigue and improve daily activities in patients.

In conclusion, the results of this study suggest that an integrated dialysis nursing intervention has a significant positive effect on fatigue among haemodialysis patients and improves their functional capacity. This study offers haemodialysis patients a more time-efficient method of exercising because the technique does not require additional time, money, or transportation. Further studies are recommended to perform randomized controlled trials to compare the effectiveness of different combinations of types of exercise, durations, implementation times, and frequencies of exercise. To date, no studies have compared these various exercise combinations. Further research should be performed on such comparisons to obtain more applicable results, and the impact of exercise on quality of life could be assessed.

The strength of this study was that the study design included a control group. The necessary biochemical markers influencing fatigue were also examined as clinical variables. Initially, it was difficult to convince patients to participate, but the compliance rate improved as they experienced the benefits of the exercise. The researcher had adequate contact time with the patients during exercise, which served as a platform to provide psychological support by addressing their concerns and issues. The limitations of this study were that long-term effects could not be observed and that a randomized control trial was not possible because of the potential contamination of the study samples. In this study, only fatigue was studied. Future studies should also examine cardiovascular status.
